# Efficacy and safety of nintedanib in patients with idiopathic pulmonary fibrosis who are elderly or have comorbidities

**DOI:** 10.1186/s12931-021-01695-y

**Published:** 2021-04-26

**Authors:** Ian Glaspole, Francesco Bonella, Elena Bargagli, Marilyn K. Glassberg, Fabian Caro, Wibke Stansen, Manuel Quaresma, Leticia Orsatti, Elisabeth Bendstrup

**Affiliations:** 1 Department of Allergy, Immunology and Respiratory Medicine, Alfred Health, and Department of Medicine, Monash University, 55 Commercial Road, Melbourne, VIC Australia; 2 Pneumology Department, Center for Interstitial and Rare Lung Disease, Ruhrlandklinik University Hospital, Essen, Germany; 3 Department of Medical Sciences, Surgery and Neurosciences, Siena University Hospital, Siena, Italy; 4 Miller School of Medicine, University of Miami, Miami, FL USA; 5 Hospital María Ferrer, Buenos Aires, Argentina; 6 Boehringer Ingelheim GmbH & Co. KG, Ingelheim Am Rhein, Germany; 7 Boehringer Ingelheim International GmbH, Ingelheim Am Rhein, Germany; 8 Department of Respiratory Diseases and Allergy, Centre for Rare Lung Diseases, Aarhus University Hospital, Aarhus, Denmark

## Abstract

**Background:**

Idiopathic pulmonary fibrosis (IPF) predominantly affects individuals aged > 60 years who have several comorbidities. Nintedanib is an approved treatment for IPF, which reduces the rate of decline in forced vital capacity (FVC). We assessed the efficacy and safety of nintedanib in patients with IPF who were elderly and who had multiple comorbidities.

**Methods:**

Data were pooled from five clinical trials in which patients were randomised to receive nintedanib 150 mg twice daily or placebo. We assessed outcomes in subgroups by age < 75 versus ≥ 75 years, by < 5 and ≥ 5 comorbidities, and by Charlson Comorbidity Index (CCI) ≤ 3 and > 3 at baseline.

**Results:**

The data set comprised 1690 patients. Nintedanib reduced the rate of decline in FVC (mL/year) over 52 weeks versus placebo in patients aged ≥ 75 years (difference: 105.3 [95% CI 39.3, 171.2]) (n = 326) and < 75 years (difference 125.2 [90.1, 160.4]) (n = 1364) (p = 0.60 for treatment-by-time-by-subgroup interaction), in patients with < 5 comorbidities (difference: 107.9 [95% CI 65.0, 150.9]) (n = 843) and ≥ 5 comorbidities (difference 139.3 [93.8, 184.8]) (n = 847) (p = 0.41 for treatment-by-time-by-subgroup interaction) and in patients with CCI score ≤ 3 (difference: 106.4 [95% CI 70.4, 142.4]) (n = 1330) and CCI score > 3 (difference: 129.5 [57.6, 201.4]) (n = 360) (p = 0.57 for treatment-by-time-by-subgroup interaction). The adverse event profile of nintedanib was generally similar across subgroups. The proportion of patients with adverse events leading to treatment discontinuation was greater in patients aged ≥ 75 years than < 75 years in both the nintedanib (26.4% versus 16.0%) and placebo (12.2% versus 10.8%) groups. Similarly the proportion of patients with adverse events leading to treatment discontinuation was greater in patients with ≥ 5 than < 5 comorbidities (nintedanib: 20.5% versus 15.7%; placebo: 12.1% versus 10.0%).

**Conclusions:**

Our findings suggest that the effect of nintedanib on reducing the rate of FVC decline is consistent across subgroups based on age and comorbidity burden. Proactive management of adverse events is important to reduce the impact of adverse events and help patients remain on therapy.

*Trial registration:* ClinicalTrials.gov NCT00514683, NCT01335464, NCT01335477, NCT02788474, NCT01979952.

**Supplementary Information:**

The online version contains supplementary material available at 10.1186/s12931-021-01695-y.

## Background

Idiopathic pulmonary fibrosis (IPF) is an interstitial lung disease characterised by progressive decline in lung function, worsening dyspnoea, and high mortality [1, 2]. IPF predominantly affects individuals over the age of 60 years [2]. Respiratory and non-respiratory comorbidities are common in patients with IPF and may impair quality of life and survival [3, 4]. Elderly patients with IPF are often frail, with multiple health problems [5], and may be less likely to be given antifibrotic therapy than younger patients [6, 7].

Nintedanib is an approved treatment for IPF, which slows the progression of the disease by reducing the rate of decline in forced vital capacity (FVC) [8, 9]. The side-effect profile of nintedanib is characterised mainly by gastrointestinal adverse events, particularly diarrhoea [8, 9]. We investigated the efficacy and safety of nintedanib in patients with IPF who were elderly and who had multiple comorbidities using pooled data from five clinical trials.

## Methods

### Trial designs

The five trials that contributed data to these analyses are described in Table 1 [8–11]. We pooled data from the periods of these trials in which patients were randomised to receive nintedanib 150 mg twice daily (bid) or placebo (52 weeks in the TOMORROW and INPULSIS trials, 12 weeks in the INMARK trial, ≥ 6 months in the Phase IIIb trial). In every trial, dose reductions from 150 mg bid to 100 mg bid and treatment interruptions were allowed to manage adverse events. The dose could be increased back to 150 mg bid after the adverse event had resolved (except during the TOMORROW trial). Specific recommendations were provided to the investigators for the management of diarrhoea and liver enzyme elevations [12].

**Table 1:** Trial designs

Trial	Key inclusion criteria	Randomised treatment	Duration
TOMORROW (NCT00514683) [8]	≥ 40 years of age FVC ≥ 50% predicted DLco 30%–79% predicted	Placebo, nintedanib 50 mg qd, nintedanib 50 mg bid, nintedanib 100 mg bid, or nintedanib 150 mg bid	Period 1: 52 weeks Period 2: Patients who completed 52 weeks’ treatment in period 1 continued treatment until the last patient had completed 52 weeks’ treatment in period 1
INPULSIS-1 and -2 (NCT01335464, NCT01335477) [9]	≥ 40 years of age FVC ≥ 50% predicted DLco 30–79% predicted	Nintedanib 150 mg bid or placebo	52 weeks
INMARK (NCT02788474) [10]	≥ 40 years of age FVC ≥ 80% predicted	Nintedanib 150 mg bid or placebo	12 weeks’ randomised treatment followed by open-label nintedanib for 40 weeks
Phase IIIb trial (NCT01979952) [11]	≥ 40 years of age FVC ≥ 50% predicted DLco 30–79% predicted	Nintedanib 150 mg bid or placebo	≥ 6 months’ randomised treatment followed by open-label nintedanib up to week 52

*bid* Twice daily, *DLco* diffusing capacity of the lung for carbon monoxide, *FVC* forced vital capacity, *qd* once daily. Patients at an increased risk of bleeding (i.e., with a genetic predisposition to bleeding, or requiring fibrinolysis, full-dose therapeutic anticoagulation, or high-dose antiplatelet therapy) and/or a recent history of thrombotic events, including stroke and transient ischaemic attacks in the previous year, myocardial infarction in the previous 6 months and unstable angina in the previous month were excluded

### Analyses

In post-hoc analyses, we assessed outcomes in subgroups of patients based on the following baseline characteristics: < 75 versus ≥ 75 years, < 5 and ≥ 5 comorbidities, Charlson Comorbidity Index (CCI) score ≤ 3 and > 3 at baseline. Thresholds of 5 comorbidities and a CCI of 3 were chosen as these were the median values in this dataset. Comorbidities were coded according to preferred terms in the Medical Dictionary for Regulatory Activities (MedDRA) version 21.1. The CCI was developed to predict one-year mortality based on age and the presence/absence of 19 comorbidities [13]. Ages of 50–59 years, 60–69 years, 70–79 years and ≥ 80 years are assigned 1, 2, 3 and 4 points, respectively. Myocardial infarction, congestive heart failure, peripheral vascular disease, dementia, chronic obstructive pulmonary disease, connective tissue disease, peptic ulcer disease, mild liver disease, and uncomplicated diabetes are assigned 1 point each; hemiplegia, moderate to severe chronic kidney disease, diabetes with end-organ damage, localised solid tumour, leukaemia, and lymphoma 2 points each; moderate to severe liver disease 3 points; and metastatic solid tumour or AIDS 6 points each. The total score ranges between 0 and 37.

The analysis set included patients who received ≥ 1 dose of nintedanib or placebo. Efficacy outcomes assessed over 52 weeks were the rate of decline in FVC (mL/year), time to first investigator-reported acute exacerbation, absolute change from baseline in St. George's Respiratory Questionnaire (SGRQ) total score, and time to death. An acute exacerbation was defined based on worsening or development of dyspnoea and the appearance of new abnormalities on high-resolution computed tomography (HRCT), with the exclusion of known causes of acute worsening in respiratory function. The SGRQ is a self-administered questionnaire comprising three domains (symptoms, activity, impact) used to assess health-related quality of life (HRQL) in patients with respiratory disease [14]. The total score ranges from 0 to 100, with higher scores indicating worse HRQL.

The rate of decline in FVC (mL/year) in subgroups was analysed using a random coefficient regression model (with random slopes and intercepts) with fixed effects for trial, treatment, sex, age and height, random effect of patient-specific intercept and time, and the interaction terms treatment-by-subgroup, time-by-subgroup and treatment-by-time-by-subgroup. The model allowed for missing data, assuming that they were missing at random; missing data were not imputed. Change from baseline in SGRQ total score was analysed using a mixed model for repeated measures (MMRM) with fixed effects for trial, treatment, visit, baseline SGRQ total score, subgroup, treatment-by-visit and baseline SGRQ total score-by-visit, and the interaction term treatment-by-subgroup; the patient effect was assumed to be random. In analyses of time to first acute exacerbation and time to death, hazard ratios and confidence intervals were obtained using a Cox’s proportional hazards model adjusted for trial, treatment, sex, age, height and subgroup and the interaction term treatment-by-subgroup. Interaction p-values were calculated to assess potential heterogeneity in the treatment effect of nintedanib versus placebo between the subgroups; interaction p-values > 0.05 were considered to indicate that no heterogeneity was detected. Analyses were not adjusted for multiplicity.

Adverse events reported by the investigators (irrespective of causality) with onset after the first dose and up to 4 weeks after the last dose of study drug were coded according to preferred terms in MedDRA (version 21.1 for analyses in subgroups by age and number of comorbidities, version 22.0 for analyses in subgroups by CCI score). Adverse events are presented descriptively.

## Results

### Subgroups by age at baseline

The analysis set comprised 1690 patients (170 from the TOMORROW trial, 1061 from the INPULSIS trials, 346 from the INMARK trial, 113 from the Phase IIIb trial). At baseline, 326 patients (19.3%) were aged ≥ 75 years. Compared with patients aged < 75 years, the subgroup aged ≥ 75 years included a greater proportion of females (25.5% vs 21.6%), had a lower mean weight (73.8 vs 80.1 kg) and body mass index (BMI) (26.6 vs 28.1 kg/m^2^), and had a lower mean FVC in mL (2634 vs 2896) but a higher mean FVC % predicted (87.7 vs 82.2) (Additional file 1: Table S1). Mean (SD) exposure to nintedanib and placebo was 9.4 (3.9) and 8.5 (4.3) months in patients aged < 75 years at baseline and 7.4 (4.4) and 6.9 (4.4) months in patients aged ≥ 75 years at baseline, respectively.

**Fig. 1:**
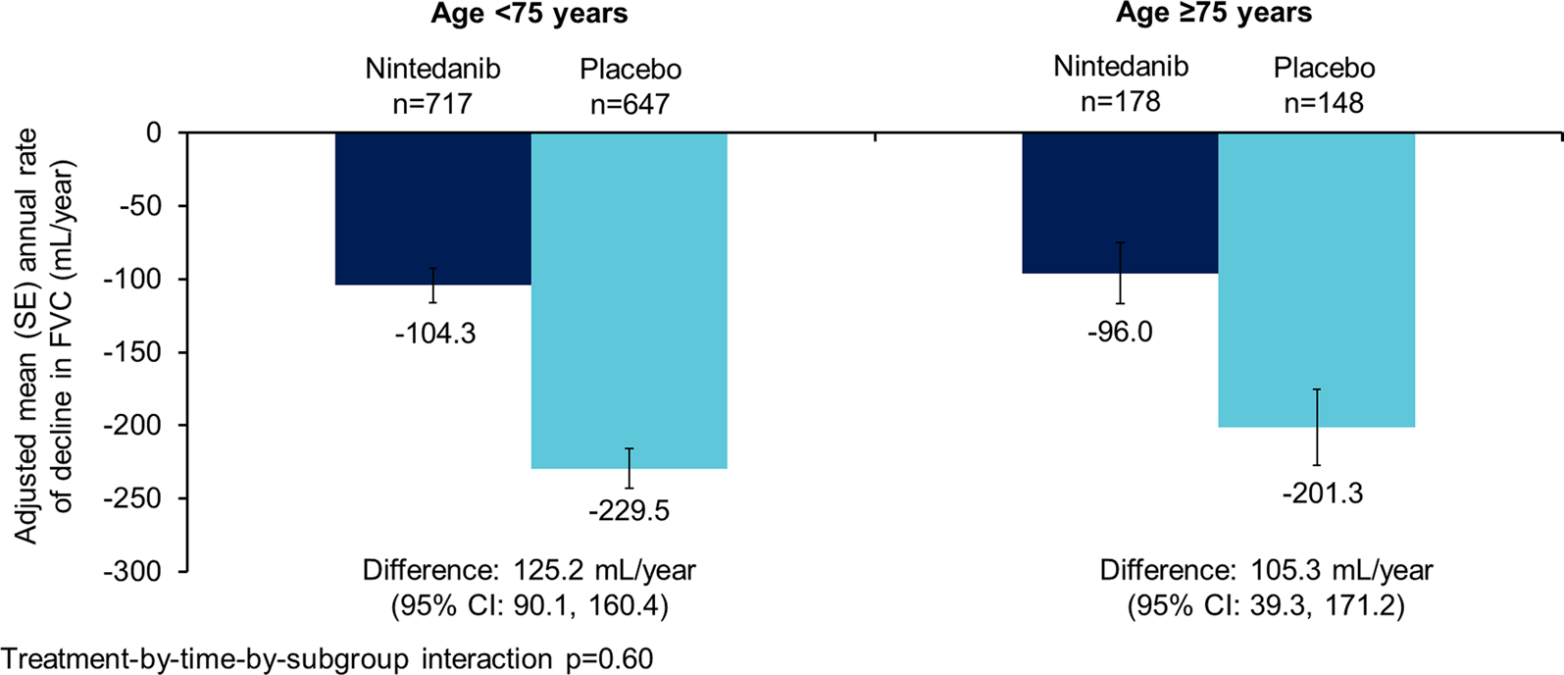
Annual rate of decline in FVC (mL/year) in subgroups by age at baseline

Nintedanib reduced the rate of decline in FVC (mL/year) versus placebo both in patients aged ≥ 75 years (difference: 105.3 [95% CI 39.3, 171.2]) and < 75 years (difference 125.2 [90.1, 160.4]) at baseline. No heterogeneity was detected in the treatment effect of nintedanib between the subgroups (p = 0.60 for treatment-by-time-by-subgroup interaction) (Fig. 1). In patients who received placebo, the increase (worsening) in SGRQ total score over 52 weeks was numerically greater in patients aged ≥ 75 years versus < 75 years at baseline (6.0 vs 3.8). The treatment effect of nintedanib on change in SGRQ total score was numerically more pronounced in patients aged ≥ 75 years versus < 75 years at baseline (− 5.2 [95%CI − 9.0, − 1.3] vs − 1.4 [− 3.0, 0.2]), but no heterogeneity was detected in the treatment effect of nintedanib between the subgroups on SGRQ total score or the individual domain scores (Table 2).

**Table 2:** Outcomes over 52 weeks in subgroups by age at baseline

	Age < 75 years		Age ≥ 75 years	
	Nintedanib (*n* = 717)	Placebo (*n* = 647)	Nintedanib (*n* = 178)	Placebo (*n* = 148)
Rate of decline in FVC (mL/year), adjusted mean (SE)	− 104.3 (11.8)	− 229.5 (13.5)	− 96.0 (20.9)	− 201.3 (26.1)
Difference (95% CI)	125.2 (90.1, 160.4)		105.3 (39.3, 171.2)	
p-value for treatment-by-time-by-subgroup interaction	0.6			
Change from baseline in SGRQ total score, adjusted mean (SE)	2.4 (0.6)	3.8 (0.6)	0.8 (1.4)	6.0 (1.6)
Difference (95% CI)	− 1.4 (− 3.0, 0.2)		− 5.2 (− 9.0, − 1.3)	
p-value for treatment-by-subgroup interaction	0.79			
Change from baseline in SGRQ symptoms score, adjusted mean (SE)	0.3 (0.9)	2.8 (1.0)	− 2.3 (1.9)	0.5 (2.4)
Difference (95% CI)	− 2.6 (− 5.1, − 0.1)		− 2.8 (− 8.7, 3.1)	
p-value for treatment-by-subgroup interaction	0.94			
Change from baseline in SGRQ activity score, adjusted mean (SE)	3.2 (0.8)	5.9 (0.9)	3.3 (1.7)	9.3 (2.1)
Difference (95% CI)	− 2.7 (− 4.9, − 0.5)		− 6.0 (− 11.2, − 0.7)	
p-value for treatment-by-subgroup interaction	0.26			
Change from baseline in SGRQ impact score, adjusted mean (SE)	2.9 (0.8)	3.7 (0.9)	2.4 (1.7)	7.2 (2.1)
Difference 95% CI)	− 0.7 (− 2.9, 1.5)		− 4.9 (− 10.1, 0.3)	
p-value for treatment-by-subgroup interaction	0.15			
Acute exacerbation of IPF, n (%)	27 (3.8)	37 (5.7)	8 (4.5)	9 (6.1)
Hazard ratio (95% CI)	0.56 (0.34, 0.93)		0.45 (0.17, 1.20)	
p-value for treatment-by-subgroup interaction	0.96			
Deaths, n (%)	33 (4.6)	32 (4.9)	10 (5.6)	14 (9.5)
Hazard ratio (95% CI)	0.78 (0.48, 1.26)		0.39 (0.17, 0.89)	
p-value for treatment-by-subgroup interaction	0.19			

*FVC* forced vital capacity, *ILD* interstitial lung disease, *SGRQ* St George’s Respiratory Questionnaire

Not all patients provided data for all endpoints; in the nintedanib and placebo groups, SGRQ total score was analysed in 682 and 619 patients aged < 75 years and 167 and 138 patients aged ≥ 75 years, SGRQ symptoms score in 697 and 624 patients aged < 75 years and 172 and 141 patients aged ≥ 75 years, SGRQ activity score in 691 and 624 patients aged < 75 years and 167 and 139 patients aged ≥ 75 years, and SGRQ impact score in 684 and 625 patients aged < 75 years and 169 and 140 patients aged ≥ 75 years, respectively

No heterogeneity was detected in the treatment effect of nintedanib in patients aged < 75 years and ≥ 75 years at baseline on time to first acute exacerbation (hazard ratio 0.56 [95% CI 0.34, 0.93] and 0.45 [0.17, 1.20], respectively; p = 0.96 for treatment-by-subgroup interaction) or time to death (hazard ratio 0.78 [95% CI 0.48, 1.26] and 0.39 [0.17, 0.89); p = 0.19 for treatment-by-subgroup interaction) (Table 2).

The adverse event profile of nintedanib was generally similar between age subgroups (Table 3). The proportion of patients who discontinued nintedanib due to adverse events was greater in patients aged ≥ 75 years than in those < 75 years at baseline (26.4% vs 16.0%). Diarrhoea led to discontinuation of nintedanib in 4.2% and 6.7% of patients aged < 75 and ≥ 75 years at baseline, respectively. Among the nintedanib-treated patients aged < 75 years (n = 426) and ≥ 75 years (n = 108) at baseline who had ≥ 1 diarrhoea adverse event, 95.3% and 93.5%, respectively, experienced events that were at worst mild or moderate in intensity. In the nintedanib and placebo groups, respectively, loperamide was taken at baseline or during treatment with trial medication by 33.2% and 5.1% of patients aged < 75 years at baseline, and by 36.0% and 9.5% of patients aged ≥ 75 years at baseline. Weight loss was reported as an adverse event by a higher proportion of patients treated with nintedanib than placebo both in patients aged < 75 years (7.9% vs 1.9%) and ≥ 75 years (14.6% vs 3.4%) at baseline. In the nintedanib and placebo groups, respectively, the mean (SD) relative change in weight at week 52 was − 3.6 (5.2) % and − 1.7 (5.1) % in patients aged < 75 years and − 5.7 (6.4) % and − 3.4 (4.9) % in patients aged ≥ 75 years at baseline.

**Table 3:** Adverse events in subgroups by age at baseline

	Age < 75 years		Age ≥ 75 years	
	Nintedanib (*n* = 717)	Placebo (*n* = 647)	Nintedanib (*n* = 178)	Placebo (*n* = 148)
Any adverse event(s)	673 (93.9)	540 (83.5)	165 (92.7)	116 (78.4)
Most frequent adverse events^a^				
Diarrhoea	426 (59.4)	132 (20.4)	108 (60.7)	22 (14.9)
Nausea	171 (23.8)	52 (8.0)	38 (21.3)	12 (8.1)
Decreased appetite	74 (10.3)	24 (3.7)	31 (17.4)	14 (9.5)
Weight decreased	57 (7.9)	12 (1.9)	26 (14.6)	5 (3.4)
Vomiting	79 (11.0)	24 (3.7)	24 (13.5)	1 (0.7)
Nasopharyngitis	84 (11.7)	87 (13.4)	22 (12.4)	15 (10.1)
Cough	90 (12.6)	82 (12.7)	13 (7.3)	18 (12.2)
Progression of IPF^b^	57 (7.9)	66 (10.2)	16 (9.0)	12 (8.1)
Serious adverse events^c^	174 (24.3)	140 (21.6)	59 (33.1)	40 (27.0)
Fatal adverse events	35 (4.9)	33 (5.1)	5 (2.8)	14 (9.5)
Any adverse event(s) leading to treatment discontinuation	115 (16.0)	70 (10.8)	47 (26.4)	18 (12.2)
Most frequent adverse events leading to treatment discontinuation^d^				
Diarrhoea	30 (4.2)	1 (0.2)	12 (6.7)	0
Progression of IPF^b^	14 (2.0)	28 (4.3)	4 (2.2)	2 (1.4)
Nausea	13 (1.8)	0	6 (3.4)	0
Decreased appetite	8 (1.1)	1 (0.2)	4 (2.2)	1 (0.7)

*IPF* idiopathic pulmonary fibrosis, *MedDRA* Medical Dictionary for Regulatory Activities

Adverse events were coded using MedDRA. Data are n (%) of patients with ≥ 1 such event

^a^Adverse events reported in > 10% of patients in any of these subgroups are shown

^b^Corresponded to MedDRA term ‘IPF’, which included disease worsening and acute exacerbations of IPF

^c^Event that resulted in death, was life-threatening, resulted in hospitalisation or prolonged hospitalisation, resulted in persistent or clinically significant disability or incapacity, was a congenital anomaly or birth defect, or was deemed serious for any other reason

^d^Adverse events that led to treatment discontinuation in > 2% of patients in any of these subgroups are shown

### Subgroups by number of comorbidities at baseline

In patients with < 5 comorbidities, the most frequent comorbidities were hypertension (27.6%), diabetes (9.8%) and gastroesophageal reflux disease (GERD) (9.6%) (Additional file 1: Table S2). In patients with ≥ 5 comorbidities, the most frequent comorbidities were hypertension (58.4%), GERD (38.8%) and hypercholesterolaemia (22.0%) (Additional file 1: Table S2). Compared with patients with < 5 comorbidities at baseline, those with ≥ 5 comorbidities included a greater proportion of females (24.6% vs 20.0%), a greater proportion of White patients (72.0% vs 52.9%), had a greater mean age (69.2 vs 65.5 years), greater mean weight (82.9 vs 75.4 kg) and BMI (28.9 vs 26.9 k/m^2^) and higher (worse) SGRQ total score (42.0 vs 37.6) (Additional file 1: Table S3). Mean (SD) exposure to nintedanib and placebo was 9.2 (4.1) and 8.1 (4.4) months in patients with < 5 comorbidities at baseline and 8.8 (4.1) and 8.2 (4.3) months in patients with ≥ 5 comorbidities at baseline, respectively.

**Fig. 2:**
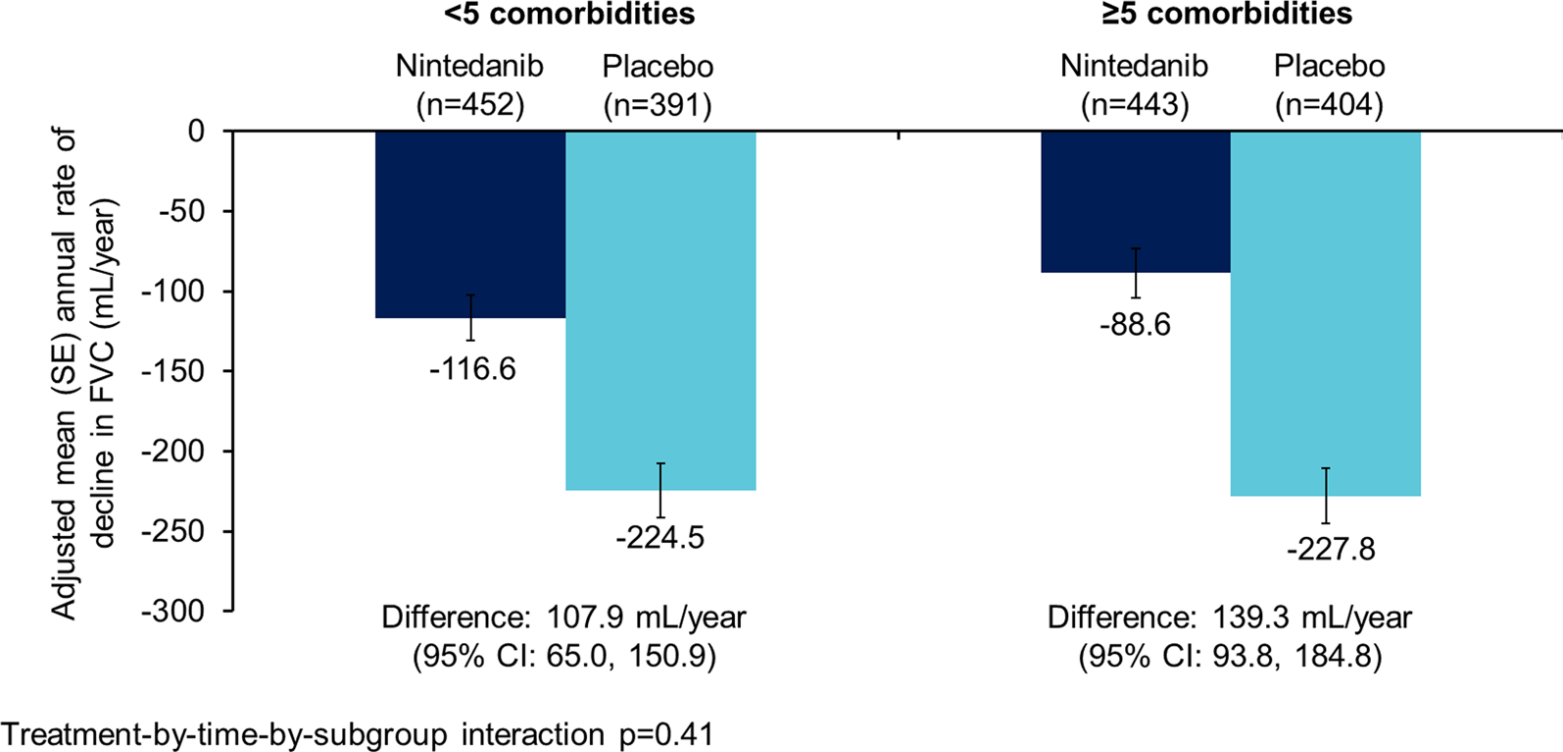
Annual rate of decline in FVC (mL/year) in subgroups by number of comorbidities at baseline

Nintedanib reduced the rate of decline in FVC (mL/year) versus placebo both in patients with < 5 comorbidities (difference: 107.9 [95% CI 65.0, 150.9]) and ≥ 5 comorbidities (difference 139.3 [93.8, 184.8]) at baseline. No heterogeneity was detected in the treatment effect of nintedanib between the subgroups (p = 0.41 for treatment-by-time-by-subgroup interaction) (Fig. 2). Over 52 weeks, SGRQ total score increased (worsened) more in patients with ≥ 5 comorbidities than in those with < 5 comorbidities at baseline in the placebo group (5.8 vs 2.6) and, to a lesser extent, in the nintedanib group (2.8 vs 1.8) (Table 4). The treatment effect of nintedanib on change from baseline in SGRQ total score was numerically more pronounced in patients with ≥ 5 comorbidities versus < 5 comorbidities at baseline (difference − 3.0 [95% CI − 5.6, − 0.5] vs − 0.8 [− 3.3, 1.8]), but no heterogeneity was detected in the treatment effect between the subgroups (p = 0.22 for treatment-by-subgroup interaction). The effect of nintedanib versus placebo on change in SGRQ symptoms score was greater in patients with ≥ 5 comorbidities than in those with < 5 comorbidities at baseline (difference − 5.0 [95% CI − 8.3, − 1.8] vs − 0.3 [− 3.5, 3.0]; p = 0.04 for treatment-by-subgroup interaction) (Table 4).

**Table 4:** Outcomes over 52 weeks in subgroups by number of comorbidities at baseline

	< 5 comorbidities		≥ 5 comorbidities	
	Nintedanib (*n* = 452)	Placebo (*n* = 391)	Nintedanib (*n* = 443)	Placebo (*n* = 404)
Rate of decline in FVC (mL/year), adjusted mean (SE)	− 116.6 (14.1)	− 224.5 (16.7)	− 88.6 (15.4)	− 227.8 (17.4)
Difference (95% CI)	107.9 (65.0, 150.9)		139.3 (93.8, 184.8)	
p-value for treatment-by-time-by-subgroup interaction	0.41			
Change from baseline in SGRQ total score, adjusted mean (SE)	1.8 (0.9)	2.6 (1.0)	2.8 (0.9)	5.8 (1.0)
Difference (95% CI)	− 0.8 (− 3.3, 1.8)		− 3.0 (− 5.6, − 0.5)	
p-value for treatment-by-subgroup interaction	0.22			
Change from baseline in SGRQ symptoms score, adjusted mean (SE)	0.8 (1.1)	1.0 (1.3)	− 1.2 (1.2)	3.8 (1.3)
Difference (95% CI)	− 0.3 (− 3.5, 3.0)		− 5.0 (− 8.3, − 1.8)	
p-value for treatment-by-subgroup interaction	0.04			
Change from baseline in SGRQ activity score, adjusted mean (SE)	2.9 (1.0)	4.6 (1.1)	3.7 (1.0)	8.2 (1.1)
Difference (95% CI)	− 1.7 (− 4.5, 1.2)		− 4.5 (− 7.4, − 1.6)	
p-value for treatment-by-subgroup interaction	0.17			
Change from baseline in SGRQ impact score, adjusted mean (SE)	1.9 (1.0)	2.3 (1.1)	3.9 (1.0)	5.9 (1.1)
Difference (95% CI)	− 0.5 (− 3.3, 2.4)		− 2.0 (− 4.9, 0.9)	
p-value for treatment-by-subgroup interaction	0.46			
Acute exacerbation of IPF, n (%)	16 (3.5)	18 (4.6)	19 (4.3)	28 (6.9)
Hazard ratio (95% CI)	0.56 (0.28, 1.10)		0.53 (0.30, 0.96)	
p-value for treatment-by-subgroup interaction	0.87			
Deaths, n (%)	17 (3.8)	21 (5.4)	26 (5.9)	25 (6.2)
Hazard ratio (95% CI)	0.51 (0.27, 0.99)		0.85 (0.49, 1.48)	
p-value for treatment-by-subgroup interaction	0.24			

*FVC* forced vital capacity, *ILD* interstitial lung disease, *SGRQ* St George’s Respiratory Questionnaire

Not all patients provided data for all endpoints; in the nintedanib and placebo groups, SGRQ total score was analysed in 430 and 373 patients with < 5 comorbidities and 419 and 384 patients with ≥ 5 comorbidities, SGRQ symptoms score in 435 and 378 patients with < 5 comorbidities and 434 and 387 patients with ≥ 5 comorbidities, SGRQ activity score in 430 and 375 patients with < 5 comorbidities and 428 and 388 patients with ≥ 5 comorbidities, and SGRQ impact score in 431 and 378 patients with < 5 comorbidities and 422 and 387 patients with ≥ 5 comorbidities, respectively

No heterogeneity was detected in the treatment effect of nintedanib in patients with < 5 comorbidities and ≥ 5 comorbidities at baseline on time to first acute exacerbation (hazard ratio 0.56 [95% CI 0.28, 1.10] and 0.53 [0.30, 0.96], respectively; p = 0.87 for treatment-by-subgroup interaction) or time to death (hazard ratio 0.51 [95% CI 0.27, 0.99] and 0.85 [0.49, 1.48); p = 0.24 for treatment-by-subgroup interaction) (Table 4).

The adverse event profile of nintedanib was generally similar between the subgroups by number of comorbidities at baseline (Additional file 1: Table S4). Serious adverse events were reported in greater proportions of patients with ≥ 5 comorbidities versus < 5 comorbidities at baseline in both the nintedanib group (29.1% vs 23.0%) and the placebo group (28.5% vs 16.6%). In the nintedanib and placebo groups, respectively, adverse events led to discontinuation of trial medication in 15.7% and 10.0% of patients with < 5 comorbidities and 20.5% and 12.1% of patients with ≥ 5 comorbidities at baseline (Additional file 1: Table S4). Diarrhoea led to discontinuation of nintedanib in 3.1% and 6.3% of patients with < 5 comorbidities and ≥ 5 comorbidities at baseline, respectively. Among the nintedanib-treated patients with < 5 comorbidities (n = 244) and ≥ 5 comorbidities (n = 290) at baseline who had ≥ 1 diarrhoea adverse event, 97.5% and 92.8%, respectively, experienced events that were at worst mild or moderate in intensity. In the nintedanib and placebo groups, respectively, loperamide was taken at baseline or during treatment with trial medication by 25.7% and 4.3% of patients with < 5 comorbidities at baseline, and by 42.0% and 7.4% of patients with ≥ 5 comorbidities at baseline.

### Subgroups by CCI score at baseline

Compared with patients with CCI score ≤ 3 at baseline, those with CCI score > 3 had a greater mean age (74.7 vs 65.4 years) and included a greater proportion of White patients (71.9% vs 59.9%) (Additional file 1: Table S5). Mean (SD) exposure to nintedanib and placebo was 9.2 (4.0) and 8.3 (4.3) months in patients with CCI score ≤ 3 at baseline and 8.2 (4.3) and 7.7 (4.4) months in patients with CCI score > 3 at baseline, respectively.

**Fig. 3:**
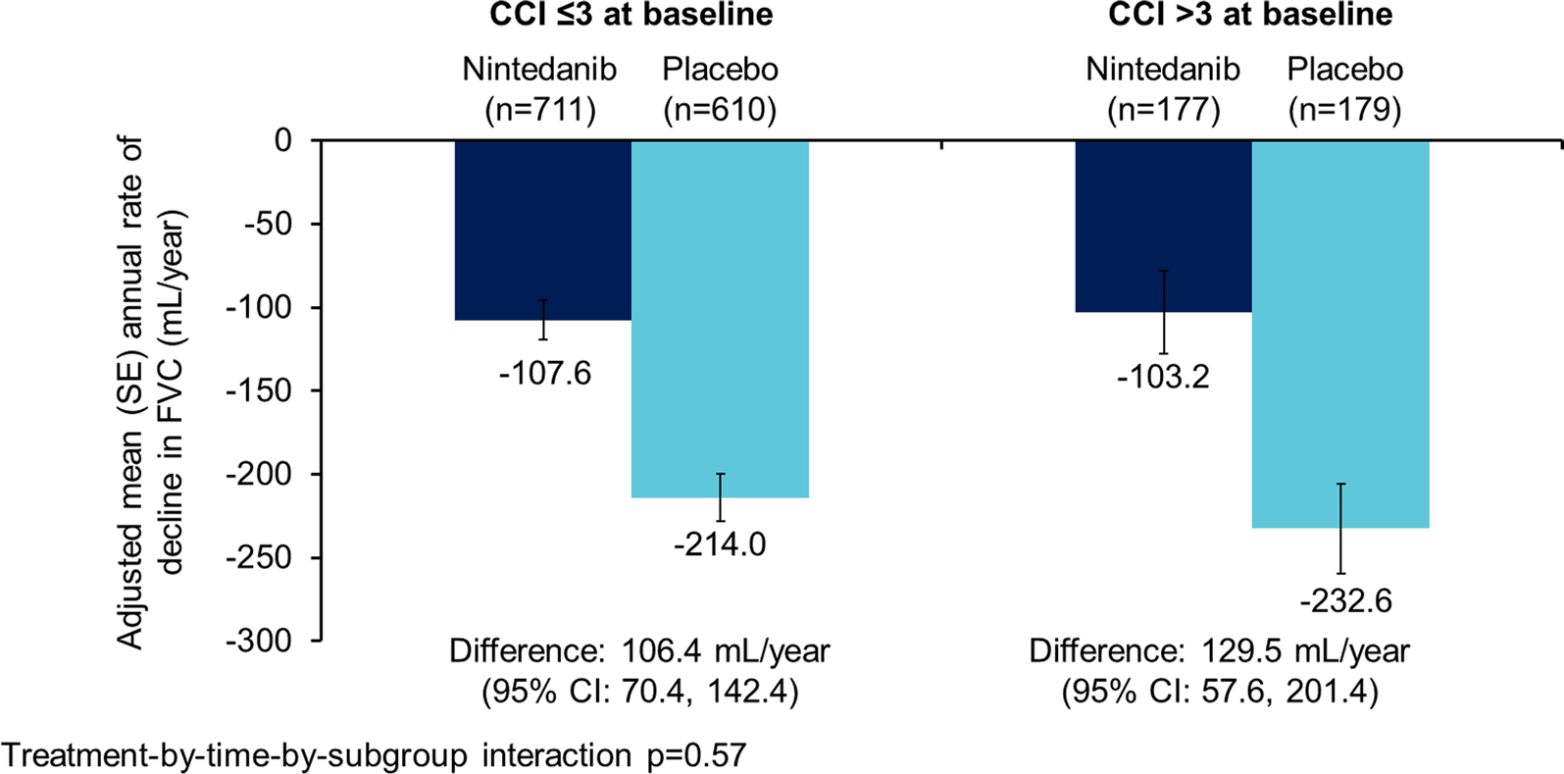
Annual rate of decline in FVC (mL/year) in subgroups by CCI score at baseline

Nintedanib reduced the rate of decline in FVC (mL/year) versus placebo both in patients with CCI score ≤ 3 (difference: 106.4 [95% CI 70.4, 142.4]) and with CCI score > 3 (difference: 129.5 [57.6, 201.4]) at baseline. No heterogeneity was detected in the treatment effect between the subgroups (p = 0.57 for treatment-by-time-by-subgroup interaction) (Fig. 3). Over 52 weeks, SGRQ total score increased (worsened) more in patients with CCI score > 3 than in those with CCI score ≤ 3 at baseline in the placebo group (6.9 vs 3.7) and to a lesser extent in the nintedanib group (3.0 vs 2.4) (Additional file 1: Table S6). The treatment effect of nintedanib on the change from baseline in SGRQ total score was numerically more pronounced in patients with CCI score > 3 than in those with CCI score ≤ 3 (difference − 3.8 [95% CI − 7.9, 0.2] vs − 1.3 [− 3.3, 0.7]), but no heterogeneity was detected in the treatment effect between the subgroups in SGRQ total score or the individual domain scores (Additional file 1: Table S6).

No heterogeneity was detected in the treatment effect of nintedanib in patients with CCI score ≤ 3 and > 3 at baseline on time to first acute exacerbation (hazard ratio 0.60 [95% CI 0.35, 1.01] and 0.49 [0.21, 1.11], respectively; p = 0.75 for treatment-by-subgroup interaction) or time to death (hazard ratio 0.69 [95% CI 0.42, 1.12] and 0.58 [0.25, 1.35); p = 0.68 for treatment-by-subgroup interaction) (Additional file 1: Table S6).

The adverse event profile of nintedanib was generally similar in both subgroups (Additional file 1: Table S7). Serious adverse events were reported in greater proportions of patients with CCI score > 3 than in those with CCI score ≤ 3 in both the nintedanib group (30.7% vs 24.9%) and placebo group (31.5% vs 20.0%). In the nintedanib and placebo groups, respectively, adverse events led to discontinuation of trial medication in 16.6% and 10.7% of patients with CCI score ≤ 3 and in 24.0% and 12.2% of patients with CCI score > 3 at baseline (Additional file 1: Table S7). Diarrhoea led to discontinuation of nintedanib in 3.9% and 7.8% of patients with CCI score ≤ 3 and > 3 at baseline, respectively. Among the nintedanib-treated patients with CCI score ≤ 3 (n = 424) and > 3 (n = 110) at baseline who had ≥ 1 diarrhoea adverse event, 94.8% and 95.5%, respectively, experienced events that were at worst mild or moderate in intensity.

## Discussion

In this analysis of pooled data from 1690 patients with IPF from five placebo-controlled trials, nintedanib had a consistent benefit in reducing the rate of FVC decline in patients aged ≥ 75 years versus < 75 years, with ≥ 5 comorbidities versus < 5 comorbidities, and with CCI score > 3 versus CCI score ≤ 3, with a safety and tolerability profile that was generally consistent across subgroups.

IPF is a disease of ageing. The incidence of IPF is greater in individuals aged > 75 years than in younger age groups [15]. In a population-based survey in Italy and the German INSIGHTS-IPF registry, over one-third of patients with IPF were aged ≥ 75 years [16, 17]. Thus the efficacy and safety of antifibrotic drugs in this patient group is of clinical relevance. We found that although the safety profile of nintedanib in patients aged ≥ 75 years was similar to that observed in younger patients, discontinuations of nintedanib due to adverse events occurred in a greater proportion of the older age group (26.4% vs 16.0%). Previous studies have also found that older patients are more likely to discontinue antifibrotic therapy [17–20]. The reasons for this are not known, but may reflect older patients being less able to tolerate the side-effects of antifibrotic therapy due to worse overall health, or to a difference in the perceived risk–benefit of treatment in older patients. Patients aged ≥ 75 years had a lower mean weight at baseline than patients aged < 75 years, and weight loss was reported as an adverse event by a higher proportion of patients in the older age group. This suggests that in the most elderly patients, clinicians need to be particularly mindful of weight loss as an adverse event and ensure that gastrointestinal events are managed effectively and nutritional interventions provided where needed [21, 22].

Most patients with IPF have multiple comorbidities. In our data set, half of the patients had ≥ 5 comorbidities at baseline. As expected based on data collected in clinical practice [3, 4], the most common comorbidities reported were hypertension, GERD, hypercholesterolaemia and diabetes. Patients with ≥ 5 comorbidities at baseline, or a CCI score > 3 at baseline, had worse HRQL (greater SGRQ total score) at baseline and a greater worsening in HRQL over the 52 weeks of follow-up. In addition, patients with ≥ 5 comorbidities at baseline, or a CCI score > 3 at baseline, were more likely to discontinue nintedanib due to adverse events. Consistent with these findings, a recent analysis of pooled data from the TOMORROW and INPULSIS trials that looked at subgroups based on the TORVAN index (a staging system derived based on age, FVC, diffusing capacity of the lung for carbon monoxide (DLco), and common comorbidities [23]) found that patients at TORVAN stage III/IV had a greater worsening of SGRQ score over 52 weeks, and were more likely to discontinue nintedanib, than patients at lower TORVAN stages [24]. These data suggest that the presence of comorbidities may complicate the management of IPF, and reinforce the importance of effective management of comorbidities as an integral part of patient care [25].

Strengths of our study include the large sample size and robust collection of data over 52 weeks. However, our analyses were post-hoc and should be considered exploratory. It should also be noted that subgroups defined by age, number of comorbidities, or CCI score differ in factors other than those parameters.

## Conclusions

These analyses of data from 1690 patients with IPF from five clinical trials suggest that the effect of nintedanib in reducing the rate of decline in FVC is consistent across patients with age ≥ 75 or < 75 years, ≥ 5 or < 5 comorbidities, and CCI score > 3 or ≤ 3 at baseline. Proactive management of adverse events through symptom management and dose adjustment is important to reduce the impact of adverse events and help patients remain on antifibrotic therapy.

A podcast of Professor Ian Glaspole discussing the data presented in this manuscript is available at: https://www.globalmedcomms.com/respiratory/glaspole/elderlypatients

## Supplementary Information

**Additional file 1: Table S1.** Baseline characteristics in subgroups by age. **Table S2** Most frequent comorbidities* at baseline in subgroups by number of comorbidities. **Table S3** Baseline characteristics in subgroups by number of comorbidities at baseline**. Table S4** Adverse events in subgroups by number of comorbidities at baseline. **Table S5** Baseline characteristics in subgroups by CCI score at baseline. **Table S6** Outcomes over 52 weeks in subgroups by CCI score at baseline. **Table S7** Adverse events in subgroups by CCI score at baseline.

## Data Availability

Researchers may request access to de-identified, analysable participant clinical study data with corresponding documentation describing the structure and content of the datasets. Upon approval, and governed by a Data Sharing Agreement, data would be shared in a secured data-access system for a period of 1 year, which may be extended upon request. Researchers should use https://trials.boehringer-ingelheim.com/ to request access to study data.

## Abbreviations

BMI: Body mass index
CCI: Charleson comorbidity index
DLco: Diffusing capacity of the lung for carbon monoxide
FVC: Forced vital capacity
GERD: Gastroesophageal reflux disease
HRCT: High-resolution computed tomography
HRQL: Health-related quality of life
IPF: Idiopathic pulmonary fibrosis
MedDRA: Medical Dictionary for Regulatory Activities
MMRM: Mixed model for repeated measures
SGRQ: St George’s Respiratory Questionnaire
