# Supplementary material for: Efficacy and safety of nintedanib in patients with idiopathic pulmonary fibrosis who are elderly or have comorbidities
Source: Respir Res. 2021 Apr 26;22:125. doi: 10.1186/s12931-021-01695-y (PMC8073950; doi:10.1186/s12931-021-01695-y)
Supplement: Supplementary file 1 — Additional file 1: Table S1. Baseline characteristics in subgroups by age. Table S2 Most frequent comorbidities* at baseline in subgroups by number of comorbidities. Table S3 Baseline characteristics in subgroups by number of comorbidities at baseline. Table S4 Adverse events in subgroups by number of comorbidities at baseline. Table S5 Baseline characteristics in subgroups by CCI score at baseline. Table S6 Outcomes over 52 weeks in subgroups by CCI score at baseline. Table S7 Adverse events in subgroups by CCI score at baseline. [file 12931_2021_1695_MOESM1_ESM.docx]

**Additional files**

**Table S1** Baseline characteristics in subgroups by age

|  | Age <75 years | | Age ≥75 years | |
| --- | --- | --- | --- | --- |
|  | Nintedanib (*n*=717) | Placebo (*n*=647) | Nintedanib (*n*=178) | Placebo (*n*=148) |
| Age, years, mean (SD) | 64.4 (6.5) | 65.1 (6.7) | 78.2 (2.9) | 78.4 (3.4) |
| Male, n (%) | 577 (80) | 493 (76) | 133 (75) | 110 (74) |
| Weight, kg, mean (SD) | 80.4 (16.4) | 79.8 (16.7) | 74.3 (14.9) | 73.2 (12.7) |
| BMI, kg/m^2^, mean (SD) | 28.2 (4.5) | 28.0 (4.6) | 26.8 (4.3) | 26.4 (3.5) |
| Race, n (%) |  |  |  |  |
| White | 433 (60) | 411 (64) | 112 (63) | 100 (67.6) |
| Asian | 211 (29) | 187 (29) | 44 (25) | 32 (22) |
| Black | 2 (<1) | 0 | 0 | 0 |
| Missing^a^ | 71 (10) | 49 (8) | 22 (12) | 16 (11) |
| Time since diagnosis, years, mean (SD) | 1.5 (1.3) | 1.4 (1.3) | 1.6 (1.4) | 1.2 (1.2) |
| Emphysema^b^, n (%) | 262 (37) | 241 (37) | 60 (34) | 45 (30) |
| Former or current smoker, n (%) | 532 (74) | 463 (72) | 119 (67) | 102 (69) |
| FVC, mL, mean (SD) | 2855 (815) | 2942 (847) | 2597 (706) | 2677 (719) |
| FVC, % predicted, mean (SD) | 80.5 (18.1) | 83.9 (19.0) | 86.7 (18.2) | 88.8 (17.4) |
| DLco, % predicted^c^, mean (SD) | 49.7 (14.4) | 52.5 (17.3) | 49.0 (14.9) | 54.0 (20.2) |
| SGRQ total score, mean (SD) | 37.5 (19.9) | 36.8 (19.0) | 40.3 (17.3) | 36.1 (20.2) |
| SGRQ symptoms score, mean (SD) | 42.4 (23.6) | 41.8 (21.9) | 44.9 (22.0) | 40.1 (22.9) |
| SGRQ activity score, mean (SD) | 49.3 (23.3) | 48.9 (23.2) | 54.0 (21.0) | 49.6 (26.0) |
| SGRQ impact score, mean (SD) | 28.6 (20.7) | 27.8 (19.9) | 30.3 (18.5) | 26.4 (20.6) |

*Abbreviations: BMI* Body mass index, *DLco* Diffusing capacity of the lungs for carbon monoxide, *FVC* Forced vital capacity.

Not all patients provided data for all variables.

^a^In France, regulation did not permit the collection of data on race.

^b^Based on qualitative assessment of high-resolution computed tomography scans by the investigators (yes or no).

^c^Corrected for haemoglobin.

**Table S2** Most frequent comorbidities* at baseline in subgroups by number of comorbidities

|  | <5 comorbidities | | ≥5 comorbidities | | |
| --- | --- | --- | --- | --- | --- |
|  | Nintedanib (*n*=452) | Placebo (*n*=391) | Nintedanib (*n*=443) | Placebo (*n*=404) |  |
| Hypertension | 131 (29.0) | 102 (26.1) | 246 (55.5) | 249 (61.6) |  |
| Gastroesophageal reflux disease | 45 (10.0) | 36 (9.2) | 170 (38.4) | 159 (39.4) |  |
| Hypercholesterolaemia | 29 (6.4) | 34 (8.7) | 94 (21.2) | 92 (22.8) |  |
| Osteoarthritis | 15 (3.3) | 19 (4.9) | 92 (20.8) | 84 (20.8) |  |
| Diabetes mellitus | 48 (10.6) | 35 (9.0) | 65 (14.7) | 48 (11.9) |  |
| Hyperlipidaemia | 19 (4.2) | 12 (3.1) | 90 (20.3) | 64 (15.8) |  |
| Benign prostatic hyperplasia | 26 (5.8) | 23 (5.9) | 57 (12.9) | 61 (15.1) |  |
| Type 2 diabetes mellitus | 19 (4.2) | 12 (3.1) | 53 (12.0) | 59 (14.6) |  |
| Sleep apnoea syndrome | 8 (1.8) | 11 (2.8) | 56 (12.6) | 60 (14.9) |  |
| Depression | 11 (2.4) | 12 (3.1) | 54 (12.2) | 52 (12.9) |  |
| Coronary artery disease | 10 (2.2) | 6 (1.5) | 50 (11.3) | 60 (14.9) |  |
| Insomnia | 13 (2.9) | 9 (2.3) | 50 (11.3) | 47 (11.6) |  |
| Hypothyroidism | 12 (2.7) | 11 (2.8) | 47 (10.6) | 40 (9.9) |  |
| Dyslipidaemia | 22 (4.9) | 9 (2.3) | 37 (8.4) | 39 (9.7) |  |
| Cough | 10 (2.2) | 10 (2.6) | 39 (8.8) | 44 (10.9) |  |
| Cataract | 10 (2.2) | 9 (2.3) | 37 (8.4) | 45 (11.1) |  |
| Osteoporosis | 13 (2.9) | 12 (3.1) | 40 (9.0) | 27 (6.7) |  |
| Seasonal allergy | 14 (3.1) | 7 (1.8) | 37 (8.4) | 33 (8.2) |  |
| Obesity | 8 (1.8) | 4 (1.0) | 39 (8.8) | 37 (9.2) |  |
| Drug hypersensitivity | 4 (0.9) | 9 (2.3) | 31 (7.0) | 37 (9.2) |  |
| Back pain | 10 (2.2) | 3 (0.8) | 36 (8.1) | 32 (7.9) |  |

Data are n (%) of subjects. Comorbidities were coded according to preferred terms in the Medical Dictionary for Regulatory Activities (MedDRA). *Comorbidities reported in ≥5% of subjects in either treatment group are shown.

**Table S3** Baseline characteristics in subgroups by number of comorbidities at baseline

|  | <5 comorbidities | | ≥5 comorbidities | |
| --- | --- | --- | --- | --- |
|  | Nintedanib (*n*=452) | Placebo (*n*=391) | Nintedanib (*n*=443) | Placebo (*n*=404) |
| Age, years, mean (SD) | 65.5 (8.2) | 65.5 (8.2) | 68.8 (7.8) | 69.6 (7.5) |
| Male, n (%) | 373 (83) | 301 (77) | 337 (76) | 302 (75) |
| Weight, kg, mean (SD) | 75.5 (14.4) | 75.2 (14.7) | 82.9 (17.2) | 83.0 (17.0) |
| BMI, kg/m^2^, mean (SD) | 27.2 (4.2) | 26.6 (3.9) | 28.8 (4.7) | 29.0 (5.0) |
| Race, n (%) |  |  |  |  |
| White | 233 (52) | 213 (54) | 312 (70) | 298 (74) |
| Asian | 186 (41) | 149 (38) | 69 (16) | 70 (17) |
| Black | 0 | 0 | 2 (<1) | 0 |
| Missing^a^ | 33 (7) | 29 (7) | 60 (14) | 36 (9) |
| Time since diagnosis, years, mean (SD) | 1.4 (1.4) | 1.3 (1.3) | 1.5 (1.3) | 1.4 (1.3) |
| Emphysema^b^, n (%) | 154 (34) | 99 (25) | 123 (28) | 86 (21) |
| Former or current smoker, n (%) | 322 (71) | 266 (68) | 329 (74) | 299 (74) |
| FVC, mL, mean (SD) | 2737 (771) | 2778 (784) | 2732 (771) | 2737 (829) |
| FVC, % predicted, mean (SD) | 79.8 (17.7) | 79.2 (17.3) | 79.2 (17.6) | 79.8 (19.1) |
| DLco, % predicted^c^, mean (SD) | 47.5 (13.5) | 47.0 (14.0) | 48.2 (13.2) | 48.4 (13.1) |
| SGRQ total score, mean (SD) | 37.3 (19.8) | 37.9 (19.4) | 42.1 (17.9) | 41.9 (17.2) |
| SGRQ symptoms score, mean (SD) | 40.6 (23.2) | 42.1 (22.6) | 48.1 (22.4) | 46.2 (21.5) |
| SGRQ activity score, mean (SD) | 48.5 (22.6) | 48.8 (22.2) | 56.3 (20.7) | 56.8 (19.8) |
| SGRQ impact score, mean (SD) | 29.5 (21.0) | 29.8 (21.1) | 31.6 (19.3) | 31.2 (19.0) |

*Abbreviations: BMI* Body mass index, *DLco* Diffusing capacity of the lungs for carbon monoxide, *FVC* Forced vital capacity

Not all patients provided data for all variables.

^a^In France, regulation did not permit the collection of data on race.

^b^Based on qualitative assessment of high-resolution computed tomography scans by the investigators (yes or no).

^c^Corrected for haemoglobin.

**Table S4** Adverse events in subgroups by number of comorbidities at baseline.

|  | <5 comorbidities | | ≥5 comorbidities | |
| --- | --- | --- | --- | --- |
|  | Nintedanib (*n*=452) | Placebo (*n*=391) | Nintedanib (*n*=443) | Placebo (*n*=404) |
| Adverse events | 410 (90.7) | 300 (76.7) | 428 (96.6) | 356 (88.1) |
| Most frequent adverse events^a^ |  |  |  |  |
| Diarrhoea | 244 (54.0) | 47 (12.0) | 290 (65.5) | 107 (26.5) |
| Nausea | 77 (17.0) | 21 (5.4) | 132 (29.8) | 43 (10.6) |
| Decreased appetite | 35 (7.7) | 12 (3.1) | 70 (15.8) | 26 (6.4) |
| Cough | 46 (10.2) | 42 (10.7) | 57 (12.9) | 58 (14.4) |
| Vomiting | 41 (9.1) | 12 (3.1) | 62 (14.0) | 13 (3.2) |
| Nasopharyngitis | 59 (13.1) | 50 (12.8) | 47 (10.6) | 52 (12.9) |
| Weight decreased | 33 (7.3) | 5 (1.3) | 50 (11.3) | 12 (3.0) |
| Bronchitis | 32 (7.1) | 26 (6.6) | 49 (11.1) | 38 (9.4) |
| Progression of IPF^b^ | 34 (7.5) | 35 (9.0) | 39 (8.8) | 43 (10.6) |
| Dyspnoea | 25 (5.5) | 30 (7.7) | 39 (8.8) | 43 (10.6) |
| Abdominal pain | 25 (5.5) | 9 (2.3) | 46 (10.4) | 17 (4.2) |
| Headache | 23 (5.1) | 13 (3.3) | 45 (10.2) | 25 (6.2) |
| Serious adverse events^c^ | 104 (23.0) | 65 (16.6) | 129 (29.1) | 115 (28.5) |
| Fatal adverse events | 14 (3.1) | 20 (5.1) | 26 (5.9) | 27 (6.7) |
| Adverse events leading to treatment discontinuation | 71 (15.7) | 39 (10.0) | 91 (20.5) | 49 (12.1) |
| Most frequent adverse events leading to treatment discontinuation^d^ |  |  |  |  |
| Diarrhoea | 14 (3.1) | 0 | 28 (6.3) | 1 (0.2) |
| Progression of IPF^b^ | 9 (2.0) | 15 (3.8) | 9 (2.0) | 15 (3.7) |
| Nausea | 4 (0.9) | 0 | 15 (3.4) | 0 |

*Abbreviations: IPF* Idiopathic pulmonary fibrosis, *MedDRA* Medical Dictionary for Regulatory Activities.

Adverse events were coded using MedDRA. Data are n (%) of patients with ≥1 such event.

^a^Adverse events reported in >10% of patients in any of these subgroups are shown

^b^Corresponded to MedDRA term ‘IPF’, which included disease worsening and acute exacerbations of IPF.

^c^Event that resulted in death, was life-threatening, resulted in hospitalisation or prolonged hospitalisation, resulted in persistent or clinically significant disability or incapacity, was a congenital anomaly or birth defect, or was deemed serious for any other reason.

^d^Adverse events leading to treatment discontinuation in >2% of patients in any of these subgroups are shown.

**Table S5** Baseline characteristics in subgroups by CCI score at baseline

|  | CCI ≤3 | | CCI >3 | |
| --- | --- | --- | --- | --- |
|  | Nintedanib (*n*=716) | Placebo (*n*=614) | Nintedanib (*n*=179) | Placebo (*n*=181) |
| Age, years, mean (SD) | 65.3 (7.5) | 65.5 (7.4) | 74.6 (6.1) | 74.7 (5.7) |
| Male, n (%) | 565 (78.9) | 460 (74.9) | 145 (81.0) | 143 (79.0) |
| Weight, kg, mean (SD) | 78.9 (15.9) | 78.3 (15.8) | 80.4 (17.7) | 79.3 (17.7) |
| BMI, kg/m^2^, mean (SD) | 28.0 (4.5) | 27.6 (4.5) | 28.0 (4.6) | 27.9 (4.4) |
| Race, n (%) |  |  |  |  |
| White | 419 (58.5) | 378 (61.6) | 126 (70.4) | 133 (73.5) |
| Asian | 219 (30.6) | 186 (30.3) | 36 (20.1) | 33 (18.2) |
| Black | 2 (0.3) | 0 | 0 | 0 |
| Missing^a^ | 76 (10.6) | 50 (8.1) | 17 (9.5) | 15 (8.3) |
| Time since diagnosis, years, mean (SD) | 1.5 (1.3) | 1.4 (1.3) | 1.6 (1.4) | 1.4 (1.3) |
| Emphysema^b^, n (%) | 229 (32.0) | 148 (24.1) | 48 (26.8) | 37 (20.4) |
| Former or current smoker, n (%) | 521 (72.8) | 435 (70.8) | 130 (72.6) | 130 (71.8) |
| FVC, mL, mean (SD) | 2812 (817) | 2933 (843) | 2770 (734) | 2757 (776) |
| FVC, % predicted, mean (SD) | 81.1 (18.2) | 84.7 (18.9) | 84.5 (18.3) | 85.4 (18.4) |
| DLco, % predicted^c^, mean (SD) | 49.4 (14.3) | 53.2 (18.2) | 49.8 (15.0) | 51.0 (17.0) |
| SGRQ total score, mean (SD) | 37.7 (19.4) | 36.1 (19.6) | 39.1 (19.4) | 38.7 (17.9) |
| SGRQ symptoms score, mean (SD) | 42.6 (23.2) | 41.0 (21.8) | 44.3 (24.0) | 43.1 (22.8) |
| SGRQ activity score, mean (SD) | 49.6 (22.7) | 47.7 (24.1) | 53.0 (23.5) | 53.6 (22.0) |
| SGRQ impact score, mean (SD) | 28.9 (20.5) | 27.4 (20.3) | 28.9 (19.5) | 27.9 (18.8) |

*Abbreviations: BMI* Body mass index, *DLco* Diffusing capacity of the lungs for carbon monoxide, *FVC* Forced vital capacity.

Not all patients provided data for all endpoints.

^a^In France, regulation did not permit the collection of data on race. Not all patients provided data for all variables.

^b^Based on qualitative assessment of high-resolution computed tomography scans by the investigators (yes or no).

^c^Corrected for haemoglobin.

**Table S6** Outcomes over 52 weeks in subgroups by CCI score at baseline

|  | CCI ≤3 | | CCI >3 | |
| --- | --- | --- | --- | --- |
|  | Nintedanib  (*n*=716) | Placebo  (*n*=614) | Nintedanib  (*n*=179) | Placebo  (*n*=181) |
| Rate of decline in FVC (mL/year), adjusted mean (SE) | -107.6 (11.9) | -214.0 (14.1) | -103.2 (24.8) | -232.6 (27.1) |
| Difference (95% CI) | 106.4 (70.4, 142.4) | | 129.5 (57.6, 201.4) | |
| p-value for treatment-by-time-by-subgroup interaction | 0.57 | | | |
| Change from baseline in SGRQ total score, adjusted mean (SE) | 2.4 (0.7) | 3.7 (0.8) | 3.0 (1.4) | 6.9 (1.5) |
| Difference (95% CI) | -1.3 (-3.3, 0.7) | | -3.8 (-7.9, 0.2) | |
| p-value for treatment-by-subgroup interaction | 0.26 | | | |
| Change from baseline in SGRQ symptoms score, adjusted mean (SE) | -0.3 (0.9) | 2.8 (1.0) | 0.4 (1.8) | 0.8 (2.0) |
| Difference (95% CI) | -3.2 (-5.8, -0.6) | | -0.4 (-5.6, 4.8) | |
| p-value for treatment-by-subgroup interaction | 0.35 | | | |
| Change from baseline in SGRQ activity score, adjusted mean (SE) | 3.2 (0.8) | 5.6 (0.9) | 4.1 (1.6) | 9.5 (1.7) |
| Difference (95% CI) | -2.4 (-4.7, -0.2) | | -5.4 (-10.0, -0.8) | |
| p-value for treatment-by-subgroup interaction | 0.26 | | | |
| Change from baseline in SGRQ impact score, adjusted mean (SE) | 2.7 (0.8) | 3.1 (0.9) | 3.5 (1.6) | 7.8 (1.7) |
| Difference (95% CI) | -0.4 (-2.7, 1.8) | | -4.2 (-8.8, 0.3) | |
| p-value for treatment-by-subgroup interaction | 0.14 | | | |
| Acute exacerbation of IPF, n (%) | 26 (3.6) | 31 (5.0) | 9 (5.0) | 15 (8.3) |
| Hazard ratio (95% CI) | 0.60 (0.35, 1.01) | | 0.49 (0.21, 1.11) | |
| p-value for treatment-by-subgroup interaction | 0.75 | | | |
| Deaths, n (%) | 34 (4.7) | 33 (5.4) | 9 (5.0) | 13 (7.2) |
| Hazard ratio (95% CI) | 0.69 (0.42, 1.12) | | 0.58 (0.25, 1.35) | |
| p-value for treatment-by-subgroup interaction | 0.68 | | | |

*Abbreviations: FVC* Forced vital capacity, *ILD* Interstitial lung disease, *SGRQ* St George’s Respiratory Questionnaire.

Not all patients provided data for all endpoints.

**Table S7** Adverse events in subgroups by CCI score at baseline

|  | CCI ≤3 | | CCI >3 | |
| --- | --- | --- | --- | --- |
|  | Nintedanib (*n*=716) | Placebo  (*n*=614) | Nintedanib (*n*=179) | Placebo  (*n*=181) |
| Adverse events | 668 (93.3) | 504 (82.1) | 170 (95.0) | 152 (84.0) |
| Most frequent adverse events^a^ |  |  |  |  |
| Diarrhoea | 424 (59.2) | 125 (20.4) | 110 (61.5) | 29 (16.0) |
| Nausea | 159 (22.2) | 50 (8.1) | 50 (27.9) | 14 (7.7) |
| Decreased appetite | 72 (10.1) | 24 (3.9) | 33 (18.4) | 14 (7.7) |
| Vomiting | 76 (10.6) | 22 (3.6) | 27 (15.1) | 3 (1.7) |
| Nasopharyngitis | 90 (12.6) | 80 (13.0) | 16 (8.9) | 22 (12.2) |
| Weight decreased | 60 (8.4) | 9 (1.5) | 23 (12.8) | 8 (4.4) |
| Cough | 86 (12.0) | 77 (12.5) | 17 (9.5) | 23 (12.7) |
| Fatigue | 43 (6.0) | 33 (5.4) | 20 (11.2) | 14 (7.7) |
| Dyspnoea | 49 (6.8) | 54 (8.8) | 15 (8.4) | 19 (10.5) |
| Progression of IPF^b^ | 58 (8.1) | 59 (9.6) | 15 (8.4) | 19 (10.5) |
| Serious adverse events^c^ | 178 (24.9) | 123 (20.0) | 55 (30.7) | 57 (31.5) |
| Fatal adverse events | 32 (4.5) | 33 (5.4) | 8 (4.5) | 14 (7.7) |
| Adverse events leading to treatment discontinuation | 119 (16.6) | 66 (10.7) | 43 (24.0) | 22 (12.2) |
| Most frequent adverse events leading to treatment discontinuation^d^ |  |  |  |  |
| Diarrhoea | 28 (3.9) | 1 (0.2) | 14 (7.8) | 0 |
| Progression of IPF^b^ | 15 (2.1) | 25 (4.1) | 3 (1.7) | 5 (2.8) |
| Nausea | 12 (1.7) | 0 | 7 (3.9) | 0 |

*Abbreviations: IPF* Idiopathic pulmonary fibrosis, *MedDRA* Medical Dictionary for Regulatory Activities

Adverse events were coded using MedDRA. Data are n (%) of patients with ≥1 such event.

^a^Adverse events reported in >10% of patients in any of these subgroups are shown.

^b^Corresponded to MedDRA term ‘IPF’, which included disease worsening and acute exacerbations of IPF.

^c^Event that resulted in death, was life-threatening, resulted in hospitalisation or prolonged hospitalisation, resulted in persistent or clinically significant disability or incapacity, was a congenital anomaly or birth defect, or was deemed serious for any other reason.

^d^Adverse events leading to treatment discontinuation in >2% of patients in any of these subgroups are shown.
